# Limitations to Work-Related Functioning of People with Persistent “Medically Unexplained” Physical Symptoms: A Modified Delphi Study Among Physicians

**DOI:** 10.1007/s10926-016-9674-x

**Published:** 2016-10-19

**Authors:** K. H. N. Weerdesteijn, F. G. Schaafsma, A. J. van der Beek, J. R. Anema

**Affiliations:** 1 Department of Public and Occupational Health, The EMGO+ Institute for Health and Care Research, VU University Medical Center (VUmc), van der Boechorststraat 7, 1081 BT Amsterdam, The Netherlands; 2 Research Center for Insurance Medicine (KCVG), AMC-UMCG-UWV-VUmc, PO Box 7057, 1007 MB Amsterdam, The Netherlands; 3 Department of Social Medical Affairs (SMZ), Dutch Social Security Agency (UWV), La Guardiaweg 94-114, 1043 DL Amsterdam, The Netherlands

**Keywords:** Medically unexplained symptoms, Insurance, disability assessment, Functional limitations, Delphi technique, Persistent physical symptoms

## Abstract

*Purpose* The purpose of this study was to obtain consensus among physicians of several medical specialties on the level of limitations to work-related functioning of people with persistent “medically unexplained” physical symptoms (PPS). *Methods* A modified Delphi study was conducted with 15 physicians of five different medical specialties. The study involved two email rounds and one meeting. In each round, the physicians prioritized the level of limitations in 78 work-related functioning items for four different PPS cases. These items were based on the Dutch Functional Ability List, national guidelines and scientific literature regarding the International Classification of Functioning. *Results* In all four cases, the physicians reached consensus on the level of limitations to work-related functioning in 49 items. The physicians reported the highest number and level of limitations for PPS of the back and lower extremities, but they reported hardly any limitations for PPS of the abdomen and genitals. For PPS of the head, they reported mainly limitations to personal and social functioning; for PPS of the neck, back and upper or lower extremities, they reported mainly limitations to dynamic movements and static postures. The physicians could not reach consensus on limitations in the category of working hours. *Conclusion* Physicians reached consensus on the level of limitations in a substantial part of work-related functioning items for PPS. There was a difference in the number and severity of limitations between different cases of PPS. The assessment of functioning seems to be based more on the specific impairment than on the disease.

**Electronic supplementary material:**

The online version of this article (doi:10.1007/s10926-016-9674-x) contains supplementary material, which is available to authorized users.

## Introduction

The most commonly used term for persistent physical symptoms that, after appropriate medical examination, lack an underlying pathological cause, and therefore cannot be fully explained by a defined organic disease, is medically unexplained physical symptoms (MUPS) [1]. Despite the term “medically unexplained”, there is more and more evidence that these physical symptoms might be explained through underlying processes and mechanism. Therefore, and on behalf of the preferences of patients, we chose to use the term Persistent “Medically Unexplained” Physical Symptoms (in short PPS) for these type of symptoms [2, 3]. PPS are common worldwide, compromising up to 50 % of all consultations in curative health care [4, 5]. Most of these symptoms are self-limiting or recover within a year after some form of therapy, but for about 20–30 % of people with PPS the symptoms persist for a longer period of time [6, 7]. Those people often have multiple, severe complaints and feel high physical distress, which mostly leads to social dysfunction [8]. In addition, the physical distress is associated with significant occupational dysfunction, long duration of sick leave, and work disability [9, 10]. For example, Hoedeman et al. reported a prevalence of 15 % of severe PPS in employees on long-term sick leave [11], and those employees may be eligible for a work disability benefit [12, 13]. In several European countries, at least 5–8 % of all new work disability benefits are awarded to people with PPS [14, 15].

The process of deciding on a claimant’s eligibility for a work disability benefit varies between countries, but what usually plays a crucial role in the assessment is the underlying cause of the complaints and the interpretation of limitations to work-related functioning [16, 17]. For the interpretation of limitations to work-related functioning, physicians have to translate medical findings and complaints to functional abilities and work disabilities [18, 19]. The outcome of such work disability assessments is not only essential for the eligibility of a work disability benefit, but also for the advice physicians give about recovery and participation opportunities, long-term prognosis, and treatment options [17, 20]. As differences in advice may form an unnecessary obstacle in the recovery and return-to-work process [12, 21], it is important there is agreement on the limitations to work-related functioning between physicians in various settings, such as insurance medicine, occupational medicine and curative health care [22, 23].

In cases of PPS, many physicians find it difficult to translate the complaints and dysfunction to limitations to work-related functioning because of the lack of objective medical findings [24, 25]. Complaints mostly do not correlate with physicians’ objective findings, nor with the work capacity rated by physicians [26]. Moreover, studies have shown that physicians of different medical specialties, and physicians from several countries, can differ in their appraisal of work ability in people with PPS [27, 28]. This emphasizes the need for more knowledge on how to assess abilities and disabilities in work for this target group, and to limit disagreements in determining eligibility for sick leave or a work disability benefit. To support physicians in these assessments, and to prevent differences in appraisals, several studies have provided recommendations and advices on standardized procedures for a work disability assessment [29, 30]. However, these recommendations are not specific in their formulation, and do not provide particular recommendations on how to translate complaints to limitations to work-related functioning.

Despite the high prevalence of people with PPS, and the high number of these people claiming a work disability benefit, there is still limited evidence for a uniform policy in the work disability assessment of people with PPS. Therefore, it is important to reach consensus between physicians of different medical specialties. The aim of this study was to reach consensus on the level of functional limitations related to work of people with PPS among physicians of several medical disciplines.

## Methods

This study used a modified Delphi technique by combining the Delphi technique and the nominal group technique [31]. These techniques are the most commonly accepted consensus methods in health care services in cases where the research evidence is incomplete, unobtainable or conflicting [32, 33]. The Delphi technique is a decision-making multistage technique among anonymous experts, whereas the nominal group technique uses structured meetings with face-to-face contact [32, 33]. Both methods aim to obtain consensus among experts on a given issue by ranking a list of items in several rounds [32, 33]. Previous research has used several modifications and combinations of these techniques as well [34, 35].

**Fig. 1:**
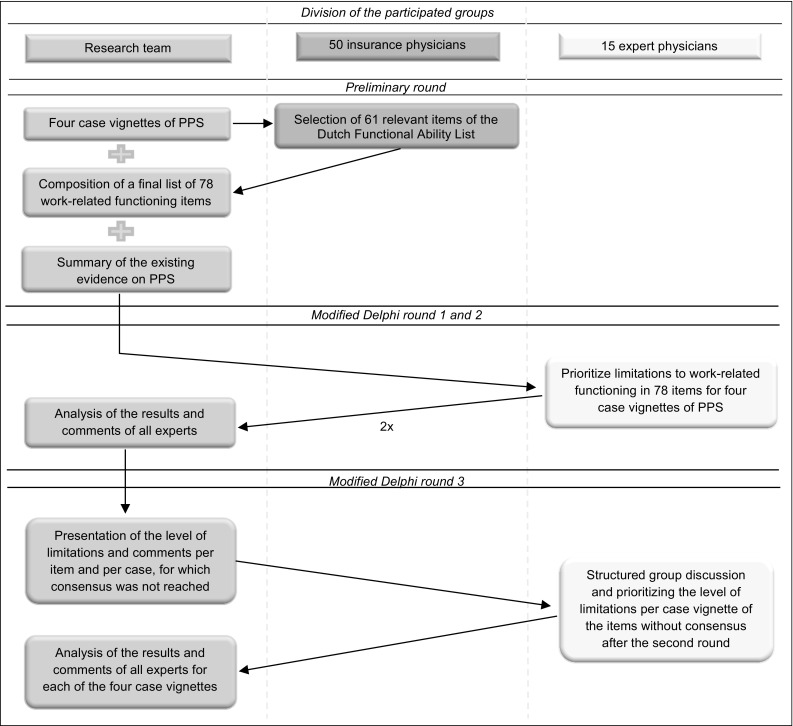
Study design

The combined Delphi technique and nominal group technique in this study was used to obtain multidisciplinary consensus among physicians on limitations in work-related functioning items for people with PPS. The entire process consisted of a preliminary round, two email rounds according to the criteria of the Delphi technique, and one meeting using the nominal group technique. An overview of the study design is presented in Fig. 1. The study was conducted between January and September 2015.

### Preliminary Round

To help provide a guide and reference points for the physicians in this study we used case vignettes of common PPS. For pragmatic reasons we chose a maximum of four case vignettes, and based the type of PPS on their prevalence according to the literature, and on the numbers of the Dutch Social Security Agency (SSA) [36, 37]. These are PPS of the head, neck and upper extremities, abdomen and/or genitals, and the back and lower extremities (Appendix A). For each type of PPS we searched the database of the SSA. We chose four real cases as case vignettes. All cases provided adequate and clear information about the health complaints, the treatment and the guidance in the return-to-work process up till now.

The Functional Ability List (FAL) [38] was used, in combination with a part of the International Classification of Functioning (ICF) [20], to develop a list of work-related functioning items. The FAL is a standardized format list of 106 functioning items, used by insurance physicians (IPs) in the Netherlands, to assess limitations and disabilities that may be important to functioning in work [38]. The FAL is comparable to the ICF, but with more detailed items. The items of the FAL are categorized into six domains: personal functioning (30 items), social functioning (17 items), dynamic movements (31 items), static postures (11 items), adjusting to environment (13 items), and working hours and time (4 items). More than two-third of the items have a dichotomous scoring option; the presence or absence of a specific functional limitation. Nearly one-third has three up to five ordinal scoring options providing a range of functional limitations [38].

In order to develop a design list of work-related functioning items for this modified Delphi study, a group of 50 IPs were asked to select which items of the FAL can give possible limitations to work-related functioning in the four different case vignettes of PPS. We then asked these IPs to recommend the medical specialty fields of physicians who should participate in this study. This was done during a meeting in which PPS, in general and in relation to work disability, were discussed between the IPs and the researcher (KW). The IPs selected 61 relevant items from the FAL, and the researchers compared this list of items with the scientific literature regarding the ICF [39–41], and with national guidelines [42] about limitations and functioning of people with PPS. As a result, 17 additional possible relevant items of work-related functioning with PPS were added to the list of 61 items. This addition resulted in a final list of 78 work-related functioning items.

### Expert Panel Selection

Based on the recommendations of the 50 IPs, this study used physicians of five medical specialties highly involved in the treatment or guidance of patients with PPS in the return-to-work process. For a structured meeting, a maximum of 20 persons is advised [31–33]. We invited 18 physicians to participate in our study, anticipating that at least 80 % would agree. The invited physicians were all considered experts in PPS because they had sufficient professional experience in dealing with PPS in their own daily practice. The resulting expert panel (n = 15) consisted of three IPs, three occupational physicians, three general practitioners, three psychiatrists, and three rehabilitation physicians.

### The Modified Delphi Technique

#### *Rounds 1 and 2*

**Fig. 2:**
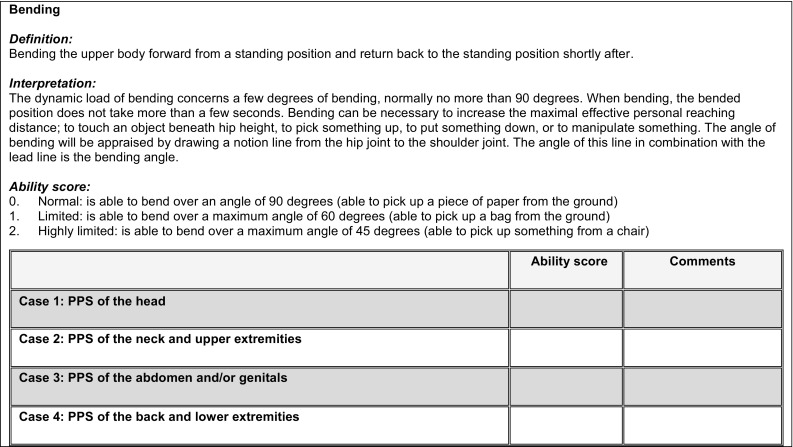
Example of one item on the work-related functioning item list

In the first round, the experts received descriptions of the four case vignettes and a summary of the existing evidence on PPS created by the researchers. In addition, they received the final list of 78 work-related functioning items, with a directory on how these items should be evaluated. The experts were asked to report any potential limitations in these items per case vignette, based on their expertise and the available literature. All experts were asked to do this independently, but they could give comments or ask questions via email. Figure 2 shows an example of one of the items to be scored.

After the first round, the researchers processed and calculated the scoring of each expert, and calculated the degree of consensus in each item per case vignette. All items with ≥75 % agreement on the level of limitations were accepted without further rating or discussion. In the second round, the level of scores per item in which no consensus was reached, together with a summary of the comments from the experts in the first round, were sent back to the experts via email. The experts were again asked to rate these items per case vignette. After this second email round, the researchers processed and calculated the results of the reported limitations in the same way as after the first round.

#### *Round 3*

In the third round, a meeting was arranged for all experts. During this meeting, the researchers explained once more the objective of the study, the results so far, and what the procedures of the nominal group technique entailed. After this introduction, the researchers graphically presented the level of limitations per item and per case vignette for which consensus was not yet reached. In addition, they presented the new comments from the second round. The experts were invited to discuss all these remaining items per case vignette. With the nominal group technique, all experts had the opportunity to clarify and to comment on the items separately. After discussing an item, the experts were asked for the last time to individually score any potential limitation of that particular item per case vignette. After the meeting, the researchers calculated the degree of consensus in this third round. This maximum of three rounds had been chosen for the modified Delhi study beforehand, for practical reasons. Consequently, the result after three rounds was the final result for the items that did not reach consensus.

## Results

Three out of 18 invited experts declined to participate in the study due to time constraints, or lack of familiarity with the FAL or Delphi technique. The 15 participating experts reported that they provided health care for at least 40 patients with PPS each year. All 15 experts responded to the first and second email rounds. Thirteen experts attended the meeting in the third round and completed the last scoring round during the meeting. The other two experts completed the third scoring round by email with input from the expert comments derived from the meeting.

## First and Second Rounds

In the first round, all experts scored the 78 items regarding limitations to work-related functioning in the four case vignettes individually. In 17 out of the 78 items, they reached consensus for all cases; these items were accepted and were not re-submitted in the next round to the experts. The experts reached consensus on 49 items for one, two or three of the cases; for 12 items they did not reach consensus in any of the cases (Table 1). All experts gave comments related to a particular item. The most frequently reported comment was the experienced difficulty in assessing the functional ability on a case vignette. The experts from curative health care experienced some difficulty in working with the items of the FAL, as they were not familiar with it. The experts with an insurance medicine background experienced difficulty accepting any limitation when no objective health problem was established. Based on these issues raised by the experts, 21 items divided over the four case vignettes were not fully assessed in this first round by one or two experts. After the second email round, there was an increase of consensus among the experts from 17 to 33 out of the 78 items for all cases. Most consensus in the cases was seen for necessary adjustments to the work environment (Table 1). A summary of the full process, rating options and results is provided in Appendix B.

**Table 1:** Number of items for which consensus on the level of limitations was reached, categorized per number of cases, per item category and per round

Category of items	Total items	Number of items with consensus, per number of cases (©)											
		Round 1				Round 2				Round 3			
		One ©	Two ©	Three ©	Four ©	One ©	Two ©	Three ©	Four ©	One ©	Two ©	Three ©	Four ©
Personal functioning	14	5	2	4	2	1	3	4	5	0	0	6	8
Social functioning	12	3	1	7	1	0	2	5	5	0	0	2	10
Dynamic movements	29	2	3	11	8	2	7	8	12	0	4	9	16
Static postures	9	0	3	3	1	1	1	3	3	1	0	2	6
Adjusting to environment	8	0	2	3	3	0	0	2	6	0	0	1	7
Working hours and time	6	0	0	0	2	1	0	1	2	1	0	1	2
Total	78	10	11	28	17	5	13	23	33	2	4	21	49

### Meeting Discussion

In the third round, during a meeting that lasted 3 h, 45 items divided over the four case vignettes were discussed. During the meeting, the comments from the experts were mostly related to the exact interpretation of a particular item, and to the translation of the provided information about medical complaints in the case vignettes to the level of limitations to work-related functioning. Several items required a more extensive discussion, which lead to consensus among the experts in the majority of cases. No agreement could be reached for the category of limitations in working hours, as the experts continued to differ in their interpretation: the IPs of the social security agency commented that they had “to use strict rules in the assessment of working hours limitations” [43], whereas all other physicians stated that “limitations in working hours can be used as a treatment, and as a tool in the return-to-work process and participation.” All experts agreed that return to work could be reached for the presented case vignettes, if the patients received proper therapy and would be provided with a healthy work environment.

### Consensus on Limitations to Work-Related Functioning

After the three modified Delphi rounds, the experts reached consensus on 49 out of 78 items (63 %) for the level of limitations to work-related functioning in all four cases. The experts did not reach consensus on any of the cases for two items that were related to the category of working hours (Table 1). The experts did reach consensus on the level of limitations to work-related functioning in 68 items (87 %) for PPS of the head, on 65 items (83 %) for PPS of the neck and upper extremities, on 72 items (92 %) for PPS of the abdomen and/or genitals, and on 64 items (82 %) on PPS of the back and lower extremities (Table 2). For the items on which the experts did reach consensus, the most frequent and the most severe limitations were found on PPS of the back and lower extremities, especially in the categories dynamic movements and static postures. For PPS of the head, the experts mostly agreed on limitations in the categories of personal and social functioning. For PPS of the abdomen and/or genitals, the experts agreed that limitations to work-related functioning would be likely for only three items. Most limitations for PPS of the neck and upper extremities were found to dynamic movements and static postures (Table 3). Appendix B lists all the items per case vignette, the level of consensus between the experts, and the degree of limitations that can be scored.

**Table 2:** Number of items for which consensus on the level of limitations was reached after the third round, categorized per case and item category

Category of items	Total items	PPS of the head	PPS of the neck and upper extremities	PPS of the abdomen and/or genitals	PPS of the back and lower extremities
Personal functioning	14	11	13	14	12
Social functioning	12	10	12	12	12
Dynamic movements	29	28	22	26	23
Static postures	9	8	7	9	7
Adjusting to environment	8	8	8	8	7
Working hours and time	6	3	3	3	3
Total	78	68	65	72	64

*PPS* persistent “medically unexplained” physical symptoms

**Table 3:** Level of limitations for which consensus was reached after the third round, categorized per case vignette and item category

PPS of the back and lower extremities		PPS of the head	
Items per category (possible limitations)	Level of limitation	Items per category (possible limitations)	Level of limitation
*Personal functioning*		*Personal functioning*	
High working tempo on the working place (2)	1^a^	Focusing attention (3)	1
Increased personal risk on the working place (2)	1	Solving problems (3)	1
*Dynamic movements*		Handling stress and other psychological demand (2)	1
Pulling or pushing (3)	2^b^	Distraction from others during work (2)	1
Lifting (4)	2	Need for predictable working situation (2)	1
Handle heavy objects frequently (2)	1	Frequent disruptions on the working place (2)	1
Turning/twisting round (2)	1	Frequent deadlines and/or production peaks (2)	1
Kneeling or squatting (2)	1	High working tempo on the working place (2)	1
Walking time per day on work (4)	2	*Social functioning*	
Walking on different surfaces (2)	1	Dealing with conflicts (3)	1
Walking stairs (4)	2	Cooperating with someone else (3)	1
Moving around using transportation (3)	1	Contact with clients (2)	1
*Static postures*		Management tasks (2)	1
Maintaining a sitting position (4)	2	*Dynamic movements*	
Maintaining a standing position (4)	2	Duration time of using a keyboard and/or mouse (4)	1
Maintaining a kneeling or squatting position (2)	1	*Adjusting to environment*	
Maintaining a bending or twisting position (2)	1	Sound intensity (2)	1
Need for possibility to change body position (2)	1	Vibration (2)	1
*Adjusting to environment*		*Working hours and working time*	
Wearing protection gear (2)	1	Working during the night (between 00:00-06:00) (2)	1
Vibration (2)	1		

PPS of the neck and upper extremities	Level of limitation	PPS of the abdomen and/or genitals	Level of limitation
Items per category (possible limitations)		Items per category (possible limitations)	
*Dynamic movements*		*Static postures*	
Turning or twisting hands or arms (2)	1	Standing time per day on work (4)	1
Reaching out (3)	1	*Adjusting to environment*	
Pulling or pushing (3)	1	Vibration (2)	1
Handle heavy objects frequently (2)	1	Possibility to use a toilet quickly (2)	1
Climbing (4)	1		
*Static postures*			
Working above shoulders (2)	1		
Maintaining head in one position (4)	1		
*Adjusting to environment*			
Wearing protection gear (2)	1		
Vibration (2)	1		

*PPS* persistent “medically unexplained” physical symptoms

^a^Mild limitations

^b^Moderate limitations

## Discussion

The main purpose of this modified Delphi study was to obtain consensus on the level of limitations in work-related functioning for workers with Persistent “medically unexplained” physical symptoms (PPS). Fifteen physicians from five different medical specialties scored the level of limitations in 78 items, based on the functional ability list (FAL) and the international classification of functioning (ICF), for four different cases of PPS. After three rounds, they obtained consensus on the level of limitations for 49 items in all four cases. The level of limitations for PPS ranged between no limitations to severe limitations, and the number and severity of limitations differed between the four PPS cases. The physicians reported the highest number and most severe limitations for PPS of the back and lower extremities, whereas they reported hardly any limitations for PPS of the abdomen and/or genitals. They reported mainly limitations in personal and social functioning for PPS of the head, and mainly limitations in dynamic movements and static postures for PPS of the back and lower extremities as well as for PPS of the neck and upper extremities. The experts did not reach consensus for any of the cases on limitations of working hours.

### Comparison with Literature

The literature on the assessment of work functioning for people with PPS is limited. As far as we know, no comparable study exists that has developed consensus-based recommendations concerning limitations to work-related functioning regarding PPS. However, previous studies have shown that for structuring functional limitations, the ICF is a useful framework for several health conditions comparable with PPS [39–41], such as chronic pain, dizziness and low back pain. For each of these conditions, a list of core items is available that describes possible limitations in bodily functions and structures. These lists address global core sets of limitations in activities, restrictions in participation and problems in environmental factors, which are essential for daily functioning. These core sets show similarities with the limitations we found in our study on dynamic movements, static postures, personal and social functioning, and environmental factors [39–41], and can be used to structure the limitations in work-related functioning on heading points. However, in comparison with our study, they are less suitable for describing and translating the precise work-related functioning [44].

Moreover, in agreement with the ICF core sets for the conditions that are comparable with PPS [39–41], our study showed, that in the assessment of work-related functioning, the different types of complaints have to be evaluated distinctly. Limitations to work-related functioning depend on the type and severity of the complaints and not on the underlying cause of the complaints, which is in contrast to other studies [45, 46].

We are not aware of any comparable studies that have developed similar recommendations for the assessment or structure of limitations in working hours for people with PPS. However, there are studies that have reported that people with PPS are able to work and participate if they are able to reduce their working hours [47], that a temporary reduction in working hours may contribute to the return-to-work process, and that it may sometimes be a better alternative than full-time sick leave [48, 49]. In addition, a reduction in working hours can be seen as part of the treatment for PPS from a curative perspective [50]. On the other hand, in many European countries a limitation of working hours provides legal ground for a work disability benefit from an insurance perspective [27, 43]. This may lead to differences in views between physicians regarding advice concerning limitations in working hours. In daily practice, this may cause conflicting advice for patients with PPS [27].

### Strength and Limitations

We believe our study had several strengths. The main strength of this study was the use of a modified Delphi technique among expert physicians of different specialties to reach consensus on limitations to work-related functioning for this difficult patient group. The broad range of medical expertise provided a wide range of competences and views that helped to maintain a broad perspective on the topic. The experts all had significant scientific credibility and/or working experience with PPS in their own specialty field. Moreover, they are representatives of their specialty field, and can therefore help improve the practical applicability of the outcomes of this study and facilitate the communication between different medical doctors. Another strength of this modified Delphi study was that all experts were provided with a summary of the relevant and available literature on this topic, and they were all able to give and revise their opinions and rates anonymously, without peer pressure. During the group discussion, they were able to discuss and elucidate their point of view. With strictly coordinated guidance from the process leader, we also tried to limit the risk of peer pressure during this discussion, however this could not be completely ruled out. Further strengths include that all participating physicians completed the entire study, and that they reached consensus on more than half of the items.

There are some weaknesses to consider when using a modified Delphi method. Firstly, the results of this study were based on the opinions of a small group of medical experts of five medical specialties. This may not be fully representative of all health care physicians. However, this is inevitable when using a qualitative approach, and using a limited number of participants was essential to having a structured meeting to reach consensus [33]. Secondly, the use of four case vignettes from the insurance medicine field as a guideline for the experts to score the limitations may also have some disadvantages, as case vignettes do not take into account co-morbidities, other medical or non-medical factors, or the information from a real medical consultation. However, case vignettes have been shown to be valuable and practical in a qualitative study such as ours [35].

Another limitation could be the use of the ICF in combination with the FAL, as not all experts were familiar with this method. The ICF is a validated list for the evaluation of functioning, and the FAL template has proven to be valuable for the assessment of functional work disabilities in the Netherlands [20, 38]. The FAL is not evidence-based, but the combination with the validated ICF makes it suitable for the purpose of this study and applicable for other European countries. IPs of the Dutch Social Security Agency have used the FAL for many years, and they therefore have much experience with using this assessment method. The other medical experts, however, were less familiar with this method, which we tried to overcome by providing full descriptions of the meanings on the items, functioning and limitations. Still, due to this knowledge lag not all items were scored by all experts in the study, which was solved in the next round with some extra information and explanation.

### Interpretation of the Results

This study indicates that, despite a small body of evidence, physicians from several medical specialties were able to reach agreement for a substantial number of limitations to work-related functioning in PPS. This deepened the insight that people with PPS can have functional limitations despite the absence of objective medical findings. On the other hand, people with PPS still have many possibilities to work functioning despite these limitations, and this study indicates that the assessment of functioning seems to be based more on the specific impairment than on the disease. Although the presented cases were all considered PPS, there was a difference in the number and severity of limitations between them, and there was also a difference in the translation from medical findings and health complaints into functional abilities and disabilities in work between the different cases of PPS. We suggest that physicians have to keep this in mind in the assessment of functional work limitations.

### Implications for Practice and Future Research

This was a first attempt to translate thoughts of physicians from different medical specialty fields to recommendations for work-related functioning related to PPS. The items in the list that reached consensus may be used in the daily practice of assessing work ability for people with PPS. This stimulates better inter-rater reliability and less conflicting advice, that may give patients with PPS a better understanding about their possibilities and work abilities. Implementing these recommendations may help the return-to-work process in the daily practice of disability assessments in the Netherlands. However, these recommendations may also be well applicable in other European countries as the issues regarding work disability in case of persistent physical symptoms are quite similar [22–26]. Besides, the insight that people with these symptoms can have functional limitations despite the absence of objective medical findings can give countries, in which people with these type of symptoms are not eligible for a disability benefit, thoughts to reconsider. As the emphasis in this study was on the items of the FAL, future studies need to assess to what extent these findings can be easily translated and are also applicable to all relevant items of the ICF.

Even though the experts reached consensus on more than half of the items in this study, there were still some items that they did not reach agreement on, especially in the category limitations in working hours. To deal with this difference in views, and to further improve inter-rater reliability, it is important to achieve further agreement among physicians, and to study the effectiveness of a temporary or permanent limitation in working hours.

## Conclusion

For four different types of PPS, physicians of five different medical specialty fields reached consensus on 49 out of 78 items on the level of limitations to work-related functioning. The physicians agreed on how to translate the health complaints of people with different cases of PPS to limitations to work-related functioning. Different cases of PPS gave different outcomes on the level of limitations to work-related functioning. Both the highest number and the most severe limitations were considered for symptoms of the back and lower extremities, especially in the dynamic and static movements categories. This means that the translation of PPS into functional limitations for work differs between different types of PPS and indicates that the assessment of functioning seems to be based more on the specific impairment than on the disease.

## Electronic supplementary material

Below is the link to the electronic supplementary material.

Supplementary material 1 (DOCX 45 kb)

Supplementary material 2 (DOCX 41 kb)
