# Supplementary material for: Limitations to Work-Related Functioning of People with Persistent “Medically Unexplained” Physical Symptoms: A Modified Delphi Study Among Physicians
Source: J Occup Rehabil. 2016 Oct 19;27(3):434–44. doi: 10.1007/s10926-016-9674-x (PMC5591343; doi:10.1007/s10926-016-9674-x)
Supplement: Supplementary file 1 — Supplementary material 1 (DOCX 45 kb) [file 10926_2016_9674_MOESM1_ESM.docx]

**Appendix A** Case vignettes

**Case 1 PPS of the head**

*Client*

The client is a 52-year-old woman, who has been sick listed for her job as shop keeper in a boutique for two years. She has a working contract for 20 hours a week.

*Personal and social status of the client*

The client is married with two grown children, who live away from home. Her husband gives her a lot of support. She is currently able to do small household chores.

*Claim of subjective limitations*

The client always feels tired and therefore cannot work for more than a few hours a day. She is not capable of working in a noisy or busy environment such as with many people, many colors, or many other stimulants. She has problems with focusing on tasks, and working under pressure or with deadlines. The client thinks that she cannot work at this moment, except for her daily chores, but maybe within one year she may be able to go back to work.

*Client history*

Two years ago the client had an accident with her bicycle. Since then, she has been suffering from many headaches, tinnitus and fatigue complaints. These complaints are present the entire day, but they may differ in intensity and duration throughout the day. At first, she was able to handle these complaints, but gradually the complaints intensified, especially in an environment with many stimulants or in stressful situations.

During the day, she tries to do some activities at home, such as her household chores or getting the groceries. This is only possible if she is able to take breaks every hour or so. She divides her daily tasks into small parts, and she will do the tasks very slowly if possible.

She sleeps a lot, but it is intermittent. She has problems with falling asleep and wakes up a couple of times during the night. In the morning, she still feels tired. During the day she is not able to focus on tasks for a long period of time because of the tiredness. After an hour of focused work, the headaches start getting worse. It feels like pressure in the head with complaints of dizziness. She has no symptoms of fainting. After two hours, she is not able to focus on her tasks anymore. To reload, she has to do some relaxing exercises or she has to stay in a quiet room.

She is not able to work because of the complaints; she is also not capable of going to social events or any other busy event anymore. She and her family have had to adjust to the new situation, for example by always turning the sound down on the television. She sometimes goes to places with her friends, but then she is not able of doing anything the next day. In the past, she was used doing a lot of things on one day, but now she is only able to do a couple of things during the day with many breaks. The few social things that she still can manage include reading magazines, watching movies with subtitles and walking with friends without talking the whole day.

*Medication*

The client uses Oxazepam, if necessary. Last week she took it twice. She stopped taking Melatonin because it did not work.

*Observational and physical examination*

The client came to the consult on her bike, and she carried a bag into the consulting room. The client said that she would not take the stairs to the first floor because she needed to save her energy. However, during the consultation she did not appear tired and she had no problems with breathing.

The client did not appear sick. She had a tidy appearance and looked like people of her own age. The client had a normal posture and condition for her age. There seemed to be no abnormalities in her movements.

*Mental status*

During the consultation, the client showed no problems with focusing or memory tasks, and there was normal eye contact. The client appeared a little bit irritated and tense, with a slight passive aggressive attitude. She was upset that she had to explain her complaints again, and mentioned that she expected that all her complaints were known already.

*Complementary examinations and treatment*

- An ear, nose and throat specialist performed an audio-logical examination, which did show a high tone loss, but not significant and suitable for her age. MRI of the cerebrum and balance tests showed no abnormalities.
- A neurologist reported a cerebral contusion. According to the client, the neurologist suggested that recovery may take one to two years, with the chance that some complaints will never get better. The neurologist had referred the client to a rehabilitation physician.
- The rehabilitation physician reported that the client had a sensory integration problem. He referred her to a rehabilitation trajectory, which is still ongoing at this moment. The trajectory consists of two hour consults three times a week, with an ergonomist, alternated with a psychologist, a physiotherapist, a movement therapist and the rehabilitation specialist. Moreover, the client has psychosomatic physiotherapist sessions with the psychologist and she does exercises to relax more. She travels to the rehabilitation center independently, taking a 20 minute bike ride. When she arrives, she does need 15 minutes to relax in a quiet room, because she cannot focus on the therapy otherwise. Between the therapy sessions she also takes 15 minutes to relax in the same manner.
- The overall goal of the trajectory is to learn how to handle stimulants better. She is already able to listen better to her body and her complaints, and to build up her activities slowly. She is also able to do more activities than before.
- She is in contact with an alternative biological center to look at her diet, which may also help to reduce the complaints.

**Case 2 PPS of the neck and upper extremities**

*Client*

The client is a 29-year-old woman, who has been sick listed for her job as a pharmacist assistant for one year. She has a working contract for 32 hours a week.

*Personal and social status of the client*

The client lives together with her boyfriend, who helps her a lot in the household chores and with lifting heavy items.

*Claim of subjective limitations*

The client has pain in her neck, and shoulder, arm and chest on the left side. Due to these health complaints, she feels less physical strength in her left arm. As a result, she is not able to lift things – for example a bag of potatoes above shoulder level – , and she is not able to open a bottle, or to wring out a cloth. In addition, she also has problems with using the keyboard on the computer, and with driving a vehicle. She says that she is not able to work due to this health complaints. The client is right handed, and with her right side she is still able to do things.

*Client history*

Two years ago the client fell on her left arm. She has had pain in her neck, and arm, shoulder and chest on the left side ever since. The left shoulder is painful the whole time and worsens when she moves her head or keeps her head in one position for a long time, for example when she works on the computer. Her left arm, from the shoulder to the elbow, feels stiff and sleepy and is especially painful with movements. The left shoulder feels like it is fixated, and it is very difficult and painful to raise her left arm above her shoulder. However, this is less difficult and painful with tape on her back from the physiotherapist. When she uses her left arm and hand for a longer period of time, it looks swollen, warm and changes color. The client’s sternum is also painful, swollen and warm, especially when moving the arms above her head and with breathing.

Because of the health complaints, she has difficulties with doing her household chores. She does not have enough strength to vacuum, clean the house, or do the dishes. She does try, but when she does this for too long or too much, her left arm becomes very painful. As a result, she cannot do any household chores anymore. The pain also creates problems with sleeping. She is not able to lie on her left side, and she awakens every two to three hours. She is awake at 7 a.m., but then still feels tired. Around 10 a.m. she gets out of bed. Throughout the rest of the day, she usually makes her bed, watches television, goes outside for a walk, does some exercises, and goes to an appointment if she has one. The client travels alone on her bike, by walking or by using the metro.

*Medication*

The client used Diclofenac, Ibuprofen and Paracetamol before; now she uses Zaldiar when she needs it for the pain. In addition, she uses Vitamin C.

*Observational and physical examination*

During the consultation, the client did not appear sick. She had a tidy appearance and looked like people of her own age. The client had a normal posture and condition for her age. There seemed to be no abnormalities in her movements. Changing from a sitting to a standing position and maintaining a sitting position did not give her any problems. The client left her left arm at rest, as much as possible during the consultation; however, she used her left hand if she needed something from her bag.

During physical examination of the neck, the client said that she felt a lot of pain with only a light touch, pressure on the neck or by movement of the neck. In addition, she reported pain and refused to put her left arm above her shoulder due to the pain. Subject to the pain, further physical examination of the upper extremities, the chest and lungs showed no abnormalities.

*Mental status*

During the consultation, the client showed no problems with focusing or memory tasks and showed no psychiatrically symptoms.

*Complementary examinations and treatment*

- The client went to the emergency room (ER) because of a swollen arm and serious pain, directly after she was fallen on her left arm. At the ER, a MRI of the neck and the left shoulder was made, which showed no pathological causes for the complaints. An X-ray of the chest and left arm showed no fractures. A physician at the ER bandaged her left arm, and gave her painkillers. The swelling of the left arm reduced after a week, but she still had a lot of pain and the color of her left arm was still changed.
- The client went to an orthopedic specialist. He advised the client to give her left arm more rest first, after which he advised the client to start with physiotherapy and Vitamin C intake. Furthermore, the orthopedic specialist sent the client to the pain specialist.
- The pain specialist prescribed other painkillers, and referred the client to the psychological care unit for individual counseling once a week. On this somatic and psychological care division the client learned to cope better with her complaints. He also advised to continue the physiotherapy to build up her physical condition, to exercise her arm muscles, to use tape if necessary to limit the pain and to stay active (twice a week). This therapy also focused on how to function in her household.
- Due to the ongoing complaints of chest pain, the client was also sent to the cardiologist, who reported no clinical pathologies of the heart, based on echo, ECG, cycle proof and blood examination.
- The client also consulted a haptonomist to learn how to bring the body and mind together.

**Case 3 PPS of the abdomen and/or genitals**

*Client*

The client is a 29-year-old woman, who has been sick listed for her job as call center assistant for two years. She has a working contract for 32-40 hours a week.

*Personal and social status of the client*

The client lives together with her boyfriend and her one-year-old son. Her boyfriend cooks and buys the groceries. Her mother helps her with the household chores and the care of her son.

*Claim of subjective limitations*

The client has a normal day and night rhythm; however, she needs a lot of help in her daily activities due to her health complaints. She does her utmost to do household chores by herself, and she tries to walk by herself. However, during the day she lies on the couch a lot because it reduces her complaints.

*Client history*

The client has pain in her abdomen, especially in the surroundings of her stomach, the entire day. Toileting is difficult because of obstipation complaints; however, two to three times a day she has stomach cramps and then she has diarrhea. At times, she feels like she is not able to do anything. Last night, she had cramps twice. Moreover, she has regurgitations, without vomiting. She cannot think of provoking factors: the complaints are not related to food or beverage. She has had a period when she felt fewer complaints, but now it is getting worse again. In the beginning, she had blood in her stool, but no slime. The pain in her abdomen worsens with movements and bumps.

*Medication*

The client used Naproxen and Macrogol in the past, but it did not help.

*Observational and physical examination*

The client came to the consult on her motor scooter. During the consultation, the client did not appear sick. She had a tidy appearance and looked like people of her own age. The client had a normal posture and condition for her age. There seemed to be no abnormalities in her movements. Maintaining a sitting position did not give her any problems, however her face revealed that she has pain.

Physical examination of the abdomen showed no serious abnormalities. Auscultation gave loudly peristalsis, but no sink murmur or souffles. Percussion and palpation were normal. The liver and spleen were not palpable, and there was no sign of rebound tenderness. However, the client felt pressure pain over the whole abdomen, especially in the upper left quadrant. The abdomen was flexible.

*Mental status*

During the consultation, the client showed no problems with focusing or memory tasks and showed no psychiatrically symptoms.

*Complementary examinations and treatment*

- The client went to a gastrointestinal and liver specialist, who conducted a gastroscopy and endoscopy because of blood in the feces and the regurgitation complaints. Both scope examinations showed no abnormalities. Pathology samples, further blood and feces examinations also showed no pathological causes for the complaints.
- The client also went to a surgeon, who did physical examinations and an ultrasound of the abdomen. Both examinations showed no abnormalities.

**Case 4 PPS of the back and lower extremities**

*Client*

The client is a 39-year-old man, who has been sick-listed for his job as shift planner for a bus company for one year. He has a working contract for 40 hours a week.

*Personal and social status of the client*

The client is single and father of four children. He lives in a house with two floors and a custom stair. He has a scoot mobile, which he uses for light groceries. He cannot drive a car anymore. Two times a week he has a maid, who helps him with the household chores. The client is in financial debt.

*Claim of subjective limitations*

The client is not able to maintain a sitting position for more than 30 minutes and a standing position for more than 5 minutes, due to his health complaints. However, if he is able to stretch out his legs, the client is able to sit for a couple of hours during the day and to stand if needed. When he moves his legs, the pain disappears for a short period of time, but the pain comes back. Walking is therefore not possible for more than 30 minutes. The client needs a walker to walk outside the house. Walking the stairs is difficult and painful. He is not able to carry heavy things or bend forward.

*Client history*

The client has had stabbing pain in his hips for a couple of years now. The pain radiates to the lower back and the legs, up to the calves and shins. The left side is worse than the right side. Sometimes, a dull feeling appears, without tingling. The complaints gradually started, and are getting worse now. Due to the pain, the client sometimes feels that he is losing his balance, and that he is going to faint. However, this has not happened before. He has no complaints of deficits.

*Medication*

The client uses Vitamin D and Amitriptyline.

*Observational and physical examination*

During the consultation, the client did not appear sick. He had a tidy appearance and looked like people of his own age. The client had a normal posture and condition for his age. However, he walked very slowly to the consultation room and he used a walker. He sat on the point of his chair to reduce the complaints of his back. However, he showed no signs of abnormal urges to move.

Physical examination of the back and hips showed, except for hyperesthesia of the lower extremities, no further abnormalities. However, the client felt pain in all movements of the hips and the back.

*Mental status*

During the consultation, the client showed no problems with focusing or memory tasks, and showed no psychiatrically symptoms. The patient pointed out that he finds it very difficult that he is so disabled at his age; he has problems with acceptance. During the consultation, he was emotional a couple of times.

*Complementary examinations and treatment*

For the complaints, the client had visit a neurologist and an orthopedic specialist. Both did examinations:

- MRI of the back and the hips showed no abnormalities.
- X-ray, vascular research and EMG of the legs showed no abnormalities.
- Examinations of the blood only showed a small decrease of Vitamin D.

The general practitioner of the client referred him to an orthopedic manual therapist. This therapist said that the vertebras were shifted and gave him an alternative treatment. However, the effect of this treatment was minimal. Thereafter, the general practitioner referred him to the department of somatology and psychology; intake has not yet taken place.
