# Supplementary material for: Limitations to Work-Related Functioning of People with Persistent “Medically Unexplained” Physical Symptoms: A Modified Delphi Study Among Physicians
Source: J Occup Rehabil. 2016 Oct 19;27(3):434–44. doi: 10.1007/s10926-016-9674-x (PMC5591343; doi:10.1007/s10926-016-9674-x)
Supplement: Supplementary file 2 — Supplementary material 2 (DOCX 41 kb) [file 10926_2016_9674_MOESM2_ESM.docx]

**Appendix B** Consensus course for all the four cases

| **Round 1;**  **Most scored level of limitations**  **(% consensus on that level)** | **Round 2;**  **Most scored level of limitations**  **(% consensus on that level)** | **Round 3;**  **Most scored level of limitations**  **(% consensus on that level)** |
| --- | --- | --- |

| **Items per category:** | Number of possible limitations | Case 1^a^ | Case 2^b^ | Case 3^c^ | Case 4^d^ | Case 1 | Case 2 | Case 3 | Case 4 | Case 1 | Case 2 | Case 3 | Case 4 |
| --- | --- | --- | --- | --- | --- | --- | --- | --- | --- | --- | --- | --- | --- |
| **Personal functioning;** |  |  |  |  |  |  |  |  |  |  |  |  |  |
| Focusing attention* | 3 | 1 (53) | 0 (100) | 0 (87) | 0 (100) | 1 (73) | - | - | - | 1 (80) | - | - | - |
| Dividing attention* | 3 | 0/1 (47) | 0 (100) | 0 (100) | 0 (100) | 1 (60) | - | - | - | 1 (60) | - | - | - |
| Insight into own abilities* | 2 | 0 (67) | 0 (87) | 0 (87) | 0 (73) | 0 (80) | - | - | 0 (87) | - | - | - | - |
| Insight into own disabilities* | 2 | 1 (53) | 0 (53) | 1 (60) | 0/1 (47) | 0 (53) | 0/1 (47) | 1 (73) | 1 (53) | 0 (93) | 0 (80) | 0 (80) | 0 (73) |
| Solving problems* | 3 | 0/1 (47) | 0 (87) | 0 (80) | 0 (60) | 1 (60) | - | - | 0 (73) | 1 (80) | - | - | 0 (100) |
| Undertaking multiple tasks* | 2 | 0/1 (47) | 0 (87) | 0 (73) | 0 (67) | 0 (53) | - | 0 (93) | 0 (80) | 0 (53) | - | - | - |
| Carrying out daily routine | 2 | 0 (93) | 0 (87) | 0 (80) | 0 (80) | - | - | - | - | - | - | - | - |
| Handling stress and other psychological demands | 2 | 1 (93) | 0 (67) | 0 (53) | 0 (60) | - | 0 (80) | 0 (53) | 0 (80) | - | - | 0 (93) | - |
| Distraction from others during work | 2 | 1 (87) | 0 (100) | 0 (100) | 0 (100) | - | - | - | - | - | - | - | - |
| Need for predictable working situation | 2 | 1 (73) | 0 (93) | 0 (80) | 0 (80) | 1 (87) | - | - | - | - | - | - | - |
| Frequent disruptions on the working place | 2 | 1 (100) | 0 (87) | 0 (73) | 0 (80) | - | - | 0 (100) | - | - | - | - | - |
| Frequent deadlines and/or frequent production peaks* | 2 | 1 (100) | 0 (67) | 0 (53) | 1 (60) | - | 0 (60) | 0 (67) | 1 (73) | - | 0 (93) | 0 (80) | 1 (73) |
| High working tempo on the working place | 2 | 1 (87) | 0 (53) | 0 (53) | 1 (67) | - | 1 (60) | 0 (60) | 1 (80) | - | 0 (60) | 0 (87) | - |
| Increased personal risk on the working place | 2 | 1 (53) | 0 (67) | 0 (87) | 0 (53) | 1 (67) | 0 (73) | - | 1 (80) | 1 (53) | 0 (93) | - | - |

| **Social functioning;** |  |  |  |  |  |  |  |  |  |  |  |  |  |
| --- | --- | --- | --- | --- | --- | --- | --- | --- | --- | --- | --- | --- | --- |
| Watching | 2 | 0 (73) | 0 (100) | 0 (100) | 0 (100) | 0 (73) | - | - | - | 0 (93) | - | - | - |
| Listening | 2 | 0 (87) | 0 (100) | 0 (100) | 0 (100) | - | - | - | - | - | - | - | - |
| Having a conversation | 3 | 0 (60) | 0 (100) | 0 (100) | 0 (100) | 1 (53) | - | - | - | 0 (87) | - | - | - |
| Handling emotional problems of others* | 3 | 1 (53) | 0 (87) | 0 (73) | 0 (73) | 1 (67) | - | 0 (80) | 0 (80) | 1 (67) | - | - | - |
| Expression of own emotions/feelings* | 3 | 0 (60) | 0 (87) | 0 (93) | 0 (80) | 0 (93) | - | - | - | - | - | - | - |
| Dealing with conflicts* | 3 | 1 (47) | 0 (80) | 0 (67) | 0 (60) | 1 (73) | - | 0 (80) | 0 (73) | 1 (100) | - | - | 0 (80) |
| Cooperating with someone else* | 3 | 1 (53) | 0 (87) | 0 (87) | 0 (87) | 1 (67) | - | - | - | 1 (80) | - | - | - |
| Contact with clients | 2 | 1 (67) | 0 (93) | 0 (100) | 0 (87) | 1 (80) | - | - | - | - | - | - | - |
| Contact with patients* | 2 | 0 (53) | 0 (80) | 0 (80) | 0 (73) | 0 (67) | - | - | 0 (80) | 0 (80) | - | - | - |
| Possibility to lean on colleagues | 2 | 0 (53) | 0 (80) | 0 (73) | 0 (67) | 1 (53) | - | 0 (87) | 0 (67) | 1 (53) | - | - | 0 (87) |
| Contact with colleagues | 2 | 0 (73) | 0 (93) | 0 (100) | 0 (100) | 0 (80) | - | - | - | - | - | - | - |
| Management tasks | 2 | 1 (73) | 0 (87) | 0 (87) | 0 (87) | 1 (93) | - | - | - | - | - | - | - |

| **Dynamic movements;** |  |  |  |  |  |  |  |  |  |  |  |  |  |
| --- | --- | --- | --- | --- | --- | --- | --- | --- | --- | --- | --- | --- | --- |
| Hand-arm use; Ball grasp | 2 | 0 (100) | 0 (80) | 0 (100) | 0 (100) | - | - | - | - | - | - | - | - |
| Hand-arm use; Pen grasp | 2 | 0 (100) | 0 (93) | 0 (100) | 0 (100) | - | - | - | - | - | - | - | - |
| Hand-arm use; Tweezer grasp | 2 | 0 (100) | 0 (100) | 0 (100) | 0 (100) | - | - | - | - | - | - | - | - |
| Hand-arm use; Key grasp | 2 | 0 (100) | 0 (93) | 0 (100) | 0 (100) | - | - | - | - | - | - | - | - |
| Hand-arm use; Cylinder grasp | 2 | 0 (100) | 0 (80) | 0 (100) | 0 (100) | - | - | - | - | - | - | - | - |
| Hand-arm use; Squeeze and grap power | 2 | 0 (100) | 0 (60) | 0 (100) | 0 (100) | - | 1 (53) | - | - | - | 0 (87) | - | - |
| Hand-arm use; Fine hand use | 2 | 0 (100) | 0 (73) | 0 (100) | 0 (100) | - | 0 (87) | - | - | - | - | - | - |
| Hand-arm use; Repetitive tasks | 2 | 0 (93) | 1 (53) | 0 (100) | 0 (100) | - | 1 (67) | - | - | - | 1 (73) | - | - |
| Using a keyboard and/or mouse | 2 | 0 (100) | 0 (73) | 0 (100) | 0 (100) | - | 0 (60) | - | - | - | 0 (93) | - | - |
| Duration time of using a keyboard and/or mouse | 4 | 0/1/2 (33) | 1 (47) | 0 (73) | 0 (67) | 1 (40) | 1 (60) | 0 (87) | 0 (60) | 1 (80) | 1 (60) | - | 0 (87) |
| Turning or twisting hands or arms | 2 | 0 (100) | 1 (100) | 0 (100) | 0 (100) | - | - | - | - | - | - | - | - |
| Reaching out* | 3 | 0 (100) | 1 (67) | 0 (100) | 0 (100) | - | 1 (87) | - | - | - | - | - | - |
| Reaching out frequently* | 4 | 0 (80) | 1 (40) | 0 (93) | 0 (80) | - | 2 (60) | - | - | - | 2 (73) | - | - |
| Bending | 3 | 0 (100) | 0 (80) | 0 (93) | 2 (47) | - | - | - | 2 (60) | - | - | - | 0 (53) |
| Bending frequently* | 4 | 0 (60) | 0 (47) | 0 (73) | 3 (47) | 0 (73) | 0 (80) | 0 (87) | 3 (67) | 0 (73) | - | - | 3 (53) |
| Turning/twisting round* | 2 | 0 (100) | 0 (73) | 0 (100) | 1 (67) | - | 0 (87) | - | 1 (80) | - | - | - | - |
| Pulling or pushing | 3 | 0 (93) | 1 (53) | 0 (80) | 1/2 (47) | - | 1 (73) | - | 2 (67) | - | 1 (100) | - | 2 (80) |
| Lifting | 4 | 0 (87) | 3 (53) | 0 (60) | 2 (47) | - | 3 (47) | 0 (60) | 2 (100) | - | 3 (47) | 0 (60) | - |
| Carrying | 4 | 0 (87) | 3 (53) | 0 (60) | 3 (47) | - | 3 (53) | 0 (73) | 3 (60) | - | 1/2 (40) | 0 (80) | 2 (67) |
| Handle light objects frequently | 4 | 0 (73) | 1/2 (33) | 0 (73) | 0 (33) | 0 (87) | 1 (53) | 0 (93) | 2 (47) | - | 1 (73) | - | 1 (53) |
| Handle heavy objects frequently | 2 | 0 (80) | 1 (93) | 0 (60) | 1 (93) | - | - | 0 (60) | - | - | - | 0 (67) | - |
| Moving head | 3 | 0 (93) | 0 (60) | 0 (100) | 0 (100) | - | 0 (53) | - | - | - | 0 (60) | - | - |
| Walking distances | 4 | 0 (93) | 0 (100) | 0 (60) | 1 (53) | - | - | 0 (67) | 1 (60) | - | - | 0 (87) | 1 (53) |
| Walking time per day on work | 4 | 0 (53) | 0 (60) | 0 (40) | 2 (47) | 0 (60) | 0 (87) | 0 (53) | 2 (100) | 0 (80) | - | 1 (53) | - |
| Walking on different surfaces | 2 | 0 (100) | 0 (93) | 0 (87) | 1 (80) | - | - | - | - | - | - | - | - |
| Walking stairs | 4 | 0 (100) | 0 (100) | 0 (80) | 2/3 (40) | - | - | - | 2 (73) | - | - | - | 2 (93) |
| Climbing* | 4 | 0 (73) | 1 (53) | 0 (67) | 3 (47) | 0 (73) | 1 (87) | 0 (87) | 3 (60) | 0 (93) | - | - | 2 (73) |
| Kneeling or squatting | 2 | 0 (100) | 0 (93) | 0 (100) | 1 (80) | - | - | - | - | - | - | - | - |
| Moving around using transportation | 3 | 0 (93) | 0 (87) | 0 (87) | 1 (60) | - | - | - | 1 (100) | - | - | - | - |

| **Static postures;** |  |  |  |  |  |  |  |  |  |  |  |  |  |
| --- | --- | --- | --- | --- | --- | --- | --- | --- | --- | --- | --- | --- | --- |
| Maintaining a sitting position | 4 | 0 (100) | 0 (93) | 0 (67) | 2 (60) | - | - | 0 (73) | 2 (80) | - | - | 0 (93) | - |
| Sitting time per day on work* | 4 | 0 (60) | 0 (60) | 0 (60) | 3 (40) | 0 (87) | 0 (67) | 0 (67) | 3 (47) | - | 0 (87) | 0 (80) | 2 (47) |
| Maintaining a standing position | 4 | 0 (93) | 0 (93) | 0 (60) | 2 (53) | - | - | 0 (87) | 2 (87) | - | - | - | - |
| Standing time per day on work | 4 | 0 (60) | 0 (53) | 0/1/2 (33) | 3 (60) | 0 (73) | 0 (53) | 1 (47) | 3 (67) | 0 (73) | 0 (73) | 1 (87) | 3 (53) |
| Maintaining a kneeling or squatting position | 2 | 0 (100) | 0 (87) | 0 (87) | 1 (100) | - | - | - | - | - | - | - | - |
| Maintaining a bending and/or twisting position | 2 | 0 (100) | 0 (53) | 0 (87) | 1 (87) | - | 1 (53) | - | - | - | 1 (53) | - | - |
| Working above shoulders | 2 | 0 (100) | 1 (100) | 0 (100) | 0 (67) | - | - | - | 0 (87) | - | - | - | - |
| Maintaining head in one position | 4 | 0 (53) | 1 (40) | 0 (93) | 0 (93) | 0 (73) | 1 (73) | - | - | 0 (93) | 1 (93) | - | - |
| Need for possibility to change body position | 2 | 0 (100) | 0 (67) | 0 (93) | 1 (80) | - | 0 (53) | - | - | - | 0 (93) | - | - |

| **Adjusting to environment;** |  |  |  |  |  |  |  |  |  |  |  |  |  |
| --- | --- | --- | --- | --- | --- | --- | --- | --- | --- | --- | --- | --- | --- |
| Temperature; Heat | 2 | 0 (93) | 0 (100) | 0 (100) | 0 (100) | - | - | - | - | - | - | - | - |
| Temperature; Cold | 2 | 0 (87) | 0 (80) | 0 (80) | 0 (60) | - | - | - | 0 (67) | - | - | - | 0 (67) |
| Draught | 2 | 0 (80) | 0 (93) | 0 (93) | 0 (73) | - | - | - | 0 (93) | - | - | - | - |
| Wearing protection gear | 2 | 0 (80) | 1 (53) | 0 (87) | 1 (67) | - | 1 (93) | - | 1 (93) | - | - | - | - |
| Sound intensity | 2 | 1 (93) | 0 (100) | 0 (100) | 0 (100) | - | - | - | - | - | - | - | - |
| Vibration | 2 | 1 (67) | 1 (80) | 1 (53) | 1 (87) | 1 (80) | - | 1 (80) | - | - | - | - | - |
| Light intensity | 2 | 0 (60) | 0 (100) | 0 (100) | 0 (100) | 0 (73) | - | - | - | 0 (100) | - | - | - |
| Possibility to use a toilet quickly | 2 | 0 (100) | 0 (100) | 1 (80) | 0 (80) | - | - | - | - | - | - | - | - |

| **Working hours and working time;** |  |  |  |  |  |  |  |  |  |  |  |  |  |
| --- | --- | --- | --- | --- | --- | --- | --- | --- | --- | --- | --- | --- | --- |
| Working during the day (between 06:00-12:00)* | 2 | 0 (87) | 0 (80) | 0 (87) | 0 (93) | - | - | - | - | - | - | - | - |
| Working during the day (between 12:00-18:00)* | 2 | 0 (87) | 0 (93) | 0 (87) | 0 (87) | - | - | - | - | - | - | - | - |
| Working during the evening (between 18:00-24:00)* | 2 | 0/1 (47) | 0 (73) | 0 (60) | 0 (53) | 0 (53) | 0 (87) | 0 (93) | 0 (80) | 0 (53) | - | - | - |
| Working during the night (between 00:00-06:00)* | 2 | 1 (60) | 0 (67) | 0 (53) | 0/1 (47) | 1 (80) | 0 (73) | 0 (73) | 1 (60) | - | 1 (53) | 1 (53) | 1 (60) |
| Possible working hours per day* | 5 | 4 (33) | 3 (33) | 3 (33) | 4 (33) | 4 (33) | 3 (53) | 3 (67) | 3 (40) | 3 (33) | 3 (33) | 3 (47) | 3 (47) |
| Possible working hours per week* | 5 | 4 (40) | 3 (40) | 3 (40) | 2/4 (27) | 4 (40) | 3 (60) | 3 (67) | 2 (40) | 3 (33) | 3 (47) | 3 (47) | 2/3 (33) |

a = Case 1; PPS of the head / b = Case 2; PPS of the neck and upper extremities / c = Case 3; PPS of the abdomen and/or genitals / d = Case 4; PPS of the back and lower extremities

* means one or two of the experts did not fill in this item for that case in one of the rounds
